# Correction: Will R&D make investors more tolerant? Analysis based on the performance forecast of Chinese listed companies

**DOI:** 10.1371/journal.pone.0317152

**Published:** 2025-01-03

**Authors:** Yixiao Chen, Yisu Wang, Huafeng Zhao, Wei Xu

There are errors in the author affiliations. The correct affiliations are as follows:

Yixiao Chen^1^, Yisu Wang^2^, Huafeng Zhao^1^, Wei Xu^1^

**1** University of Jinan, Jinan, Shandong Province, China, **2** Renmin University of China, Beijing, China
