# Supplementary material for: Will R&D make investors more tolerant? Analysis based on the performance forecast of Chinese listed companies
Source: PLoS One. 2023 Jan 26;18(1):e0280237. doi: 10.1371/journal.pone.0280237 (PMC9879430; doi:10.1371/journal.pone.0280237)
Supplement: Attachment — Submitted filename: Response to Reviewers.docx [file pone.0280237.s002.docx]

Dear Prof. Fiaschetti and Reviewers:

Thank you for your letter and the reviewers’ comments concerning our manuscript. These comments are valuable and very helpful. We have read through the comments carefully and have made corrections. Based on the instructions provided in your letter, we uploaded the file of the revised manuscript. Revisions in the text are shown using yellow highlighting, and the responses to the reviewer's comments are presented as follows.

We are grateful to you for allowing us to resubmit a revised copy of the manuscript, and we highly appreciate your time and consideration.

Sincerely.

Wei Xu.

**Response to the reviewer's comments:**

**Reviewer #1:**

This paper explores the impact of corporate R&D on investors' short-term performance expectations by analyzing listed companies that disclosed quantitative performance forecasts from 2016 to 2021 in the Shanghai and Shenzhen A-share markets. The results suggest that corporate R&D investment significantly reduces investors' short-term performance expectations that is, investors have a significant tolerance effect on enterprises with higher R&D investment.

**Response:** Thank you very much for the positive comments and constructive suggestions. Please find the following detailed responses to your comments and suggestions.

**Q1.** It is not clear what is the problem at hand. How this work differs from the previous work done in this area? It needs sharp contextualization.

**Response:** Thank you very much for your comment, which is highly appreciated. The previous research on the market reaction of R&D is mostly based on the assumption of the complete rationality of investors and risk aversion under the framework of traditional financial theory. Our work analyzes this problem based on prospect theory under the assumption of limited rationality of investors, which is a new research perspective. This point was not well discussed in the previous manuscript, so we revised the relevant parts in the introduction and literature review.

**Q2.** This paper takes all A-share listed companies that disclosed their performance forecasts from 2016 to 2021 as samples. Contextualization from the context of sample firms and the country is missing. Why do authors rely on Chinese firms only? The authors should add a paragraph on the dynamics and importance of focusing on this sample.

**Response:** Thank you for your constructive suggestion. As a typical emerging capital market, China's capital market is characterized by a high proportion of individual investors, which also means that the overall rationality of investors is relatively low. At the same time, with the development of innovation in China, the guidance and investment of China's capital market for innovation are also very obvious. Therefore, if we explore the impact of corporate R&D on investors from the perspective of bounded rationality, China's capital market will be a very representative sample. In the introduction, we added a background introduction and corresponding discussion and discussed the effectiveness and limitations of using this sample in the discussion section.

**Q3.** Make the literature section comparable that how your work is superior to previous work. Also, no theoretical underpinnings are provided in the literature section and empirical literature included in the paper does not cover recent evidence.

**Response:** Thank you for your kind suggestions. The question of how our work is superior to previous work has been answered in question 1, so it will not be repeated. We have added a literature review on prospect theory and adjusted the logic of the overall literature review. In addition, we have supplemented the recent empirical literature.

**Q4.**The study applied a multiple regression model to analyse the data. How it is well suited for achieving the study’s objectives. The authors should add a significant justification for why you picked this estimation model.

**Response:** Thank you for your kind question. In this study, the cumulative abnormal return of the performance forecast is used as the proxy variable of investors' short-term performance expectations, so the part of the cumulative abnormal return affected by the performance change information must be controlled. The traditional event study method is that the study of a single variable has difficulty meeting the needs of this study, so we choose to use a multiple regression model. We have added a corresponding discussion to the multiple regression model.

**Q5.**It is good that the authors have employed robustness analysis with different models and measurements of variables. However, the economic significance and discussion of the main results as well as of the robustness analysis is missing. The findings and discussion section does not provide the needed explanation and support from previous studies. The key findings should be compared with the existing studies, and they should highlight whether these findings are consistent or the other way around.

**Response:** Thank you for your kind suggestions. In the research results section, we added a discussion of the economic significance of the empirical results (including the robustness test), and in the research results and conclusions sections, we added a comparison between the key research findings and existing research results.

**Q6.**Also, I encourage authors to develop the practical implications specifically based on the findings of the study instead of general discussion. Authors need to be more specific when it comes to possible future extensions of their work.

**Response:** Thank you for your constructive suggestion. We divided the conclusion and discussion into two sections, and in the discussion section, we provided a deeper discussion of the research results of this paper (including the conditions for the generation of the tolerance effect, as well as the applicability and limitations of the results). In addition, we have also revised the part related to future work to make it more specific.

**Q7.**There are grammatical mistakes in the paper. Please carefully go through the paper for in-text citation errors, spelling and grammatical mistakes before submitting the revised version.

**Response:** We are very sorry for our incorrect writing. To address these issues, we found a professional organization to edit the manuscript.

Reviewer #2:

The topic of the manuscript is interesting. Studying the behavior of investors and the factors that influence this behavior is important in terms of identifying barriers to economic development. However, the manuscript could be improved, my comments are attached:

**Q1.** it is necessary to clarify the research methods used in the abstract, also describe the general conclusion based on the results (in the context of significance)

**Response:** Thank you very much for your constructive suggestion. The abstract is truly important for a paper. We added general conclusions to the abstract and described the research methods in more detail.

**Q2.**it is recommended to revise the structure of the Introduction, focusing on a consistent description of the relevance, scientific and practical problems, bright points of view on this problem (other studies) with a transition to the purpose of the study and tasks, study design. The authors also need to clarify the choice of China as a field for empirical research (in terms of relevance and the possibility of applying the results to other countries)

**Response:** Thank you for your kind suggestions, which are highly appreciated. We made a comprehensive adjustment to the structure of the introduction to better lead to the purpose and innovation of this study. In addition, in the introduction, we also added the background of the Chinese sample and the reasons for its selection, and in the discussion, we added an analysis of the applicability and limitations of the results.

**Q3.** section 2.3 Research hypotheses is closer to 3. Methodology than to 2. Literature review

**Response:** Thank you for your kind suggestions. We referred to some other papers of the same type published on PLOS One and found that many of them put the literature review and research hypothesis in the same section. Therefore, we retained the original framework and changed the title of the second part to “Literature review and research hypotheses”.

**Q4.**authors are advised to separate the Discussion section and the Conclusions section, therefore section 5 needs to be revised

**Response:** Thank you for your constructive suggestion. We split section 5 according to the suggestions and performed a more profound analysis of the research results in the discussion section.

**Q5.** a clear clarification of research limitations and assumptions is required (including making a reasonable conclusion about the possibility of extrapolating the results for other countries, and not just for China)

**Response:** Thank you for your kind suggestion. In the discussion section, we added an analysis of the applicability and limitations of the research conclusions and discussed the possibility of applying the tolerance effect to the capital markets of other countries.

**Q6.**the data file could be improved for reader accessibility (use English). It is recommended to add notes for headings with symbols; also add sheet descriptions and sheet titles.

**Response:** We apologize for our negligence in the accessibility of our data. We reorganized the data files, deleted unnecessary sheets, and used all English. Unclear variable names have been modified to make them consistent with the paper. We also added a title to the chart.
