# Supplementary material for: Will R&D make investors more tolerant? Analysis based on the performance forecast of Chinese listed companies
Source: PLoS One. 2023 Jan 26;18(1):e0280237. doi: 10.1371/journal.pone.0280237 (PMC9879430; doi:10.1371/journal.pone.0280237)
Supplement: Attachment — Submitted filename: Response to Reviewers.docx [file pone.0280237.s003.docx]

Dear Prof. Fiaschetti and reviewers:

Thank you for your letter and the reviewers’ comments concerning our manuscript. These new comments were very helpful in making the paper more rigorous. We have read through the comments carefully and have made corrections. Based on the instructions provided in your letter, we uploaded the file of the revised manuscript. The revisions made to the text are highlighted in yellow, and the responses to the reviewers' comments are presented as follows.

We are grateful that you have allowed us to resubmit a revised copy of the manuscript, and we greatly appreciate your time and consideration.

Sincerely,

Wei Xu

**Response to the reviewers' comments:**

**Reviewer #2:**

The authors did a great job and provided a revised manuscript, taking into account the comments of the reviewers. However, there are a few items that could be improved (minor revision):

**Response:** Thank you very much for the positive comments and constructive suggestions. Please find the following detailed responses to your comments and suggestions.

**Q1.** In Section 2.3, the authors gave a description of the H1 hypothesis, but did not provide a description of the H2 hypothesis.

**Response:** Thank you very much for your comment; this section was not rigorous enough. Therefore, we added a description of H2.

**Q2.** Authors need to provide links to sources of primary data. In section 3.1, the authors provide data resources, but no references.

**Response:** Thank you for your constructive suggestion. We added links to our sources of primary data in section 3.1.

**Q3.** Authors are advised to clarify the sample size. "This paper takes all A-share listed companies that disclosed their performance forecasts from 2016 to 2021 as samples." How many such companies?

**Response:** Thank you for your kind question. When we checked this section, we found that there was a problem with the description of the sample size (we forgot to modify it when updating the sample). Therefore, we corrected the error and described the sample size in more detail.

**Q4:** Since a sample has some specific characteristics that determine its quality, researchers may be interested in the question of the coverage of the dissemination of the results. The authors are advised to briefly clarify the representativeness of the results (through reliability indicators, calculation of sampling error, etc., if applicable in this case).

**Response:** Thank you for your kind suggestions. We also believe that the quality of samples has a decisive impact on the reliability and representativeness of research results. Therefore, we used the Heckman model in section 4.3.3 to reduce the impact of sample selection bias on the research results. The regression results of the Heckman model show that selection bias has no significant impact on our sample quality. To explain this point more clearly, we added a description in section 4.3.3.

**Reviewer #3:**

This is an interesting paper. All suggestions are carefully fixed and I recommend to publish this interesting paper.

**Response:** Thank you very much for your recognition of our work.
